# Orthostatic Hypotension: A Prodromal Marker of Parkinson's Disease?

**DOI:** 10.1002/mds.28303

**Published:** 2020-09-23

**Authors:** Lisanne J. Dommershuijsen, Alis Heshmatollah, Francesco U.S. Mattace Raso, Peter J. Koudstaal, M. Arfan Ikram, M. Kamran Ikram

**Affiliations:** ^1^ Department of Epidemiology Erasmus MC University Medical Center Rotterdam the Netherlands; ^2^ Department of Neurology Erasmus MC University Medical Center Rotterdam the Netherlands; ^3^ Department of Geriatric Medicine Erasmus MC University Medical Center Rotterdam the Netherlands

**Keywords:** orthostatic hypotension, Parkinson's disease, autonomic dysfunction, prodromal, general population

## Abstract

**Background:**

Orthostatic hypotension is common in patients with Parkinson's disease (PD). However, it remains unknown whether orthostatic hypotension is a marker of prodromal PD or more advanced disease. The objectives of this study were to assess whether orthostatic hypotension is a prodromal marker of PD in the general population.

**Methods:**

This study was embedded in the Rotterdam Study, a large prospective population‐based cohort in the Netherlands. We measured orthostatic hypotension in 6910 participants. First, we determined the relation between prevalent PD and orthostatic hypotension using logistic regression. Second, we followed PD‐free participants for the occurrence of PD until 2016 and studied the association between orthostatic hypotension and the risk of PD using Cox proportional hazards models. All models were adjusted for age and sex.

**Results:**

At baseline, the mean age ± standard deviation of the study population was 69.0 ± 8.8 years, and 59.1% were women. Orthostatic hypotension was present in 1245 participants (19.8%), and 62 participants (1.0%) had PD at the time of orthostatic hypotension measurement. Participants with PD were significantly more likely to have orthostatic hypotension (odds ratio, 1.88; 95% confidence interval, 1.09–3.24). During a median (interquartile range) follow‐up of 16.1 years (8.5–22.7 years), 122 participants were diagnosed with incident PD. Orthostatic hypotension at baseline was not associated with an increased risk of PD (hazard ratio, 0.97; 95% confidence interval, 0.59–1.58).

**Conclusions:**

Our study suggests that orthostatic hypotension is common in patients with PD, but that orthostatic hypotension is not associated with an increased risk of PD and thus is not a prodromal marker of PD in the general population. © 2020 The Authors. *Movement Disorders* published by Wiley Periodicals LLC on behalf of International Parkinson and Movement Disorder Society

Parkinson's disease (PD) is a complex neurodegenerative disorder involving both motor and nonmotor symptoms. The prodromal phase of PD can start as early as 20 years prior to diagnosis and is dominated by nonmotor signs including constipation, rapid eye movement sleep behavioral disorder, and hyposmia.^1^, ^2^, ^3^, ^4^ Orthostatic hypotension has also been described as a common nonmotor sign of PD,^5^, ^6^ but was typically attributed to a later stage of disease.^2^

Recent studies have suggested that orthostatic hypotension can already develop in the prodromal phase of PD.^1^ Determining whether orthostatic hypotension should be considered a prodromal marker of PD is important because prodromal markers allow earlier recognition of PD and could help to identify individuals eligible for neuroprotective trials. Furthermore, early recognition and treatment of orthostatic hypotension and other nonmotor symptoms of PD is required because these symptoms negatively impact quality of life.^7^, ^8^

The association between orthostatic hypotension and the risk of PD has been limitedly studied so far. Some small studies have shown an increased risk of PD in participants with orthostatic hypotension.^9^, ^10^, ^11^ Also, a large registry‐based study has demonstrated that participants with hypotension, including orthostatic hypotension, carried an increased risk of PD.^12^ However, the association between orthostatic hypotension and PD has not been prospectively studied in a large sample of the general population yet.

Thus, we aimed to identify whether orthostatic hypotension in the general population is a prodromal marker of PD. Hence, we determined the association between prevalent PD and orthostatic hypotension and the association between orthostatic hypotension and the risk of incident PD in a general population.

## Methods

### Study Population

**FIG. 1:**
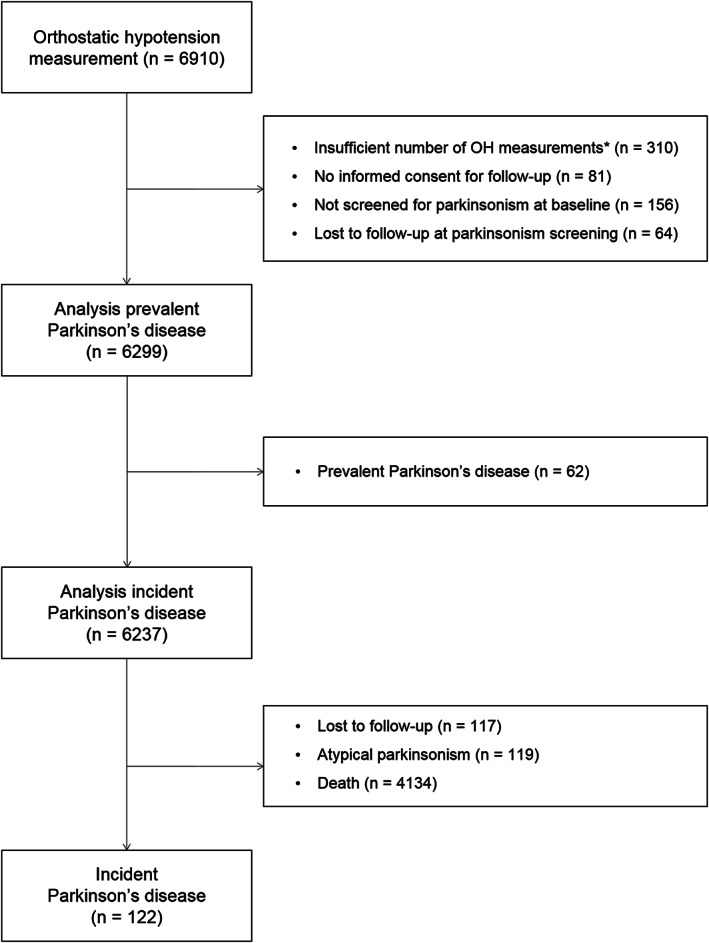
Flowchart of study participants. *Participants were excluded if the following measurements were missing: both the systolic and diastolic blood pressure measurements in supine position, all systolic or all diastolic blood pressure measurements in the standing position, or all heart rate measurements.

This study is embedded in the Rotterdam Study, a large prospective population‐based cohort study in the Netherlands.^13^ We included the first cohort in the current study, which was initiated in 1990 and consisted of 7983 participants aged 55 years and older. Participants were interviewed at home and examined at the research center from October 1989 to July 1993. In total, 6910 participants visited the research center and underwent examination for orthostatic hypotension. We included 6299 participants in the prevalent analyses and 6237 participants in the incident analyses; reasons for exclusion can be found in Figure 1.

The Rotterdam Study has been approved by the Medical Ethics Committee of the Erasmus MC University Medical Center and by the review board of the Ministry of Health, Welfare, and Sports of the Netherlands according to the Population Study Act Rotterdam Study. All participants provided written informed consent to participate in the study.

### Assessment of Orthostatic Hypotension

Blood pressure and heart rate (HR) were measured using an automatic recorder (Dinamap, Critikon). Baseline blood pressure was defined as the mean of 2 measurements on the upper right arm with the participant in the supine position after 5 minutes of rest. The measurements were repeated in a standing position after 1, 2, 3, 4, and 5 minutes. Orthostatic hypotension was measured once at baseline and was defined as a decrease in systolic blood pressure (SBP) of at least 20 mm Hg or a decrease in diastolic blood pressure (DBP) of at least 10 mm Hg within 3 minutes after standing up from the supine position, in accordance with the consensus statement of the American Academy of Neurology.^14^ To define neurogenic orthostatic hypotension, we calculated the difference in HR and SBP in the supine position and after 3 minutes of standing. If the ratio between the difference in HR and SBP (ΔHR/ΔSBP) was lower than 0.5 in participants with orthostatic hypotension, we classified participants as having neurogenic orthostatic hypotension.^15^ In addition, we excluded all participants with diabetes, cancer, renal failure (eGFR ≤ 15), and stroke as possible causes of neurogenic orthostatic hypotension.^18^ Furthermore, we studied early and delayed orthostatic hypotension, defined as orthostatic hypotension within 1 minute and between 3 and 5 minutes, respectively. Participants with orthostatic hypotension were divided into asymptomatic and symptomatic based on the question they were asked directly after the measurement whether they had felt unwell within the minutes following postural change.

### Parkinson's Disease Ascertainment

At study entry, we used a 2‐phase design to identify participants with parkinsonism (PS), an approach that has been previously described in more detail.^16^ In short, participants were evaluated for self‐reported PS, the use of antiparkinsonian drugs, and signs of PS at neurologic screening. Participants who screened positive for this first phase were invited for a structured clinical examination by a research physician with expertise in neurological disorders to establish parkinsonism, which included neurological examination (including motor examination by the Unified Parkinson's Disease Rating Scale), gathering of medical history, and attainment of additional information from medical records of general practitioners and specialists.

During follow‐up, we used 4 overlapping modalities to continuously detect potential cases of PS: continuous monitoring of clinical records and antiparkinsonian medication use, self‐reporting of PD, and in‐person screening at the research center (on average every 4 years). Medical records of all persons who screened positive in any of these methods were studied further, and case reports were evaluated by a panel led by an experienced neurologist.

Parkinsonism was defined as the presence of hypo‐ or bradykinesia and at least 1 of the following cardinal signs: resting tremor, rigidity, or postural imbalance, as observed by any physician; or a clinical diagnosis of PS by a neurologist or geriatrician. PD was diagnosed after exclusion of atypical PS, which was defined as parkinsonism associated with preexistent dementia, use of antidopaminergic drugs, cerebrovascular disease, multiple system atrophy, progressive supranuclear palsy, and other rare causes (eg, corticobasal degeneration). PD entailed the criteria of PSand at least 1 of the following: (1) a clinical PD diagnosis by a neurologist or geriatrician; (2) a positive response to dopaminergic treatment; or (3) dopamine transporter scan findings consistent with PD. After the initial diagnosis, medical records of all incident PS cases (both PD and atypical PS) continued to be scrutinized until the end of the study period for new information that could lead to a revision of the diagnosis. The time at risk for PD in this study ended at the first of the following: diagnosis of incident PD, loss to follow‐up, death, or January 1, 2016. The completeness of follow‐up for PD, according to person‐years,^17^ was 98.7%. Loss to follow‐up did not differ between participants with orthostatic hypotension and those without orthostatic hypotension.

### Statistical Analysis

We performed imputations for missing data on SBP, DBP, and HR. The maximum proportion of missing information of the imputed variables was 6.4%. A detailed overview of the missing information can be found in Table S1. Five imputations were performed using age, sex, prevalent and incident PD, unwell feeling during the examination, and other available blood pressure and HR variables as predictors. We determined the relation of prevalent PD with orthostatic hypotension using a logistic regression model adjusted for age and sex. Subsequently, we excluded participants who had prevalent PD at baseline (n = 62), resulting in 6237 participants for the survival analysis. We assessed the association between the presence of orthostatic hypotension and the risk of incident PD using Cox proportional‐hazards models adjusted for age and sex. We censored death and loss to follow‐up in all analyses. In addition, we determined the relation between orthostatic hypotension and death by constructing Kaplan‐Meier curves.

To further examine the time relation of the association between orthostatic hypotension and incident PD, we repeated the prospective analysis with shorter follow‐up times (5, 10, and 15 years). As a sensitivity analysis, we performed the analyses for prevalent and incident PD using a modified definition of orthostatic hypotension in participants with hypertension.^18^ In this definition, participants with hypertension (SBP ≥ 150 mm Hg or DBP ≥ 90 mm Hg) required a larger decrease in blood pressure to meet the criteria for orthostatic hypotension, that is, a decrease in SBP of at least 30 mm Hg or a decrease in DBP of at least 15 mm Hg within 3 minutes after standing up from the supine position. In addition, we repeated the analyses for neurogenic orthostatic hypotension using a heart rate increase of less than 15 beats per minute as the cutoff for neurogenic orthostatic hypotension.^18^ Finally, we performed a complete case analysis to determine the effect of the imputations on our results.

All analyses were performed using R version 3.6.2.

## Results

The baseline characteristics of participants who underwent orthostatic hypotension examination are presented in Table 1. At baseline, the mean age ± standard deviation (SD) of participants without prevalent PD was 68.9 ± 8.8 years, and 3690 participants (59.2%) were women. In total, 62 participants (1.0%) had PD at baseline. Participants with prevalent PD were older (77.4 ± 8.5 years) and included relatively fewer women (35 [56.5%]). Information about the diagnosis date was available for 52 of the 62 participants with prevalent PD. Age at diagnosis of PD was on average 73.5 ± 9.9 years, and participants with prevalent PD were diagnosed on average 3.7 ± 4.9 years before the orthostatic hypotension measurement. Mean ± SD Hoehn and Yahr score, available for 47 participants, was 2.3 ± 1.1.

**TABLE 1:** Characteristics of study population at baseline

	No prevalent PD (n = 6237)	Prevalent PD (n = 62)
Age (y)	68.9 ± 8.8	77.4 ± 8.5
Female	3690 (59.2%)	35 (56.5%)
Systolic blood pressure supine (mm Hg)	142 ± 21	145 ± 22
Diastolic blood pressure supine (mm Hg)	73 ± 12	71 ± 12
Heart rate supine (beats per minute)	72 ± 12	73 ± 10
Blood pressure‐lowering medication use^a^	1954 (31.3%)	25 (40.3%)
Beta‐blocker medication use^a^	892 (14.3%)	6 (9.7%)
Atrial fibrillation^a^	289 (4.6%)	5 (8.1%)
Orthostatic hypotension	1220 (19.6%)	25 (40.3%)
Neurogenic orthostatic hypotension	633 (10.1%)	13 (21.0%)
Symptomatic orthostatic hypotension	167 (2.7%)	8 (12.9%)
Early orthostatic hypotension, within 1 min	826 (13.2%)	20 (32.3%)
Delayed orthostatic hypotension, after 3 minutes	290 (4.6%)	2 (3.2%)

Data are presented as frequency (percentage) for categorical values and mean ± standard deviation for continuous variables.

^a^ Information on blood pressure and beta‐blocker medication use was missing in 4 participants (0.1%) without prevalent PD, and none was missing in participants with prevalent PD. Information on atrial fibrillation was missing in 208 participants (3.3%) without prevalent PD and in 1 participant (1.6%) with prevalent PD.

Orthostatic hypotension was present in 1220 participants without prevalent PD (19.6%) and in 25 participants with prevalent PD (40.3%). Individuals with prevalent PD were significantly more likely to have orthostatic hypotension (OR, 1.88; 95% CI, 1.09–3.24; Table 2). The strongest association was found for symptomatic orthostatic hypotension (OR, 3.34; 95% CI, 1.52–7.35), although the confidence interval of this association was wide because of a small number of PD patients with symptomatic orthostatic hypotension (n = 8). Individuals with PD were also more likely to have early orthostatic hypotension (OR, 1.86; 95% CI, 1.04–3.33). The association between PD and neurogenic orthostatic hypotension was not statistically significant (OR, 1.50; 95% CI, 0.79–2.84). The odds ratio for neurogenic orthostatic hypotension did not change substantially when participants with diabetes, cancer, renal failure, and stroke were excluded from the analyses (OR, 1.53; 95% CI, 0.78–2.98). Delayed orthostatic hypotension could not be included in the prevalent analysis because of a too‐small sample of participants with prevalent PD and delayed orthostatic hypotension (n = 2).

**TABLE 2:** Association of prevalent Parkinson's disease with orthostatic hypotension

	Orthostatic hypotension	Neurogenic OH	Symptomatic OH	Early OH
No prevalent Parkinson's disease				
Number of OH participants/all participants	1220/6237	633/6237	167/6237	826/6237
Prevalent Parkinson's disease				
Number of OH participants/all participants	25/62	13/62	8/62	20/62
Odds ratio (95% CI)	**1.88 (1.09–3.24)**	1.50 (0.79–2.84)	**3.34 (1.52**–**7.35)**	**1.86 (1.04–3.33)**

CI, confidence interval; OH, orthostatic hypotension. All models are adjusted for age and sex.

After a median (interquartile range) follow‐up of 16.1 years (8.5–22.7 years), 122 participants were diagnosed with incident PD. The median time to PD within these 122 participants was 7.2 years (3.3–15.1 years). Mean ± SD age at incident PD diagnosis was 78.8 ± 6.6 years, and 63 participants with incident PD were women (51.6%). Orthostatic hypotension at baseline was not associated with an increased risk of PD (HR, 0.97; 95% CI, 0.59–1.58; Table 3). There was also no association between neurogenic orthostatic hypotension, early orthostatic hypotension, or delayed orthostatic hypotension at baseline and the risk of PD. Exclusion of participants with comorbidities that could explain the presence of neurogenic orthostatic hypotension did not change the hazard ratio for neurogenic orthostatic hypotension (HR, 0.82; 95% CI, 0.38–1.78). Symptomatic orthostatic hypotension could not be included in the incident analysis because of a too‐small sample of participants with symptomatic orthostatic hypotension developing incident PD (n = 3). Finally, we found increased risk of death in participants with orthostatic hypotension and neurogenic orthostatic hypotension compared with participants without orthostatic hypotension (Figure S1).

**TABLE 3:** Orthostatic hypotension and risk of incident Parkinson's disease

	Incident Parkinson's disease	
	n/N	Hazard ratio (95% CI)
No orthostatic hypotension	100/5017	1.0 (reference)
Orthostatic hypotension	22/1220	0.97 (0.59–1.58)
No neurogenic orthostatic hypotension	112/5604	1.0 (reference)
Neurogenic orthostatic hypotension	10/633	0.82 (0.39–1.70)
No early orthostatic hypotension	110/5411	1.0 (reference)
Early orthostatic hypotension	12/826	0.74 (0.40–1.38)
No delayed orthostatic hypotension	115/5947	1.0 (reference)
Delayed orthostatic hypotension	7/290	1.23 (0.62–2.40)

CI, confidence interval; n, number of participants with Parkinson's disease; N, total number of participants. All models are adjusted for age and sex.

Repeating the analysis with different cutoffs for follow‐up time did not yield different results. We found no association between orthostatic hypotension and the risk of PD if the follow‐up time was restricted to 5 years (HR, 1.06; 95% CI, 0.53–2.14), 10 years (HR, 1.13; 95% CI, 0.63–2.02), or 15 years (HR, 0.93; 95% CI, 0.53–1.63); see Table 4. The sensitivity analyses using a modified orthostatic hypotension definition for participants with hypertension attenuated the association between prevalent PD and orthostatic hypotension, neurogenic orthostatic hypotension, and early orthostatic hypotension, but strengthened the association between PD and symptomatic orthostatic hypotension. The results for incident PD remained consistent (Tables S2 and S3). When we defined neurogenic orthostatic hypotension using the cutoff of 15 beats per minute in heart rate, we found that prevalent PD was significantly associated with neurogenic orthostatic hypotension (OR, 2.15; 95% CI, 1.24–3.73). This adapted definition of neurogenic orthostatic hypotension did not change the results of the prospective analyses. The complete case analysis made the odds ratios for the analysis with prevalent PD somewhat more pronounced, but did not substantially change the results for incident PD (data not shown).

**TABLE 4:** Orthostatic hypotension and risk of Parkinson's disease with increasing years of follow‐up (5, 10, and 15 years)

	Follow‐up ≤ 5 years		Follow‐up ≤ 10 years		Follow‐up ≤ 15 years	
	n/N	Hazard ratio (95% CI)	n/N	Hazard ratio (95% CI)	n/N	Hazard ratio (95% CI)
No orthostatic hypotension	33/5017	1.0 (reference)	57/5017	1.0 (reference)	73/5017	1.0 (reference)
Orthostatic hypotension	11/1220	1.06 (0.53–2.14)	18/1220	1.13 (0.63–2.02)	18/1220	0.93 (0.53–1.63)

CI, confidence interval; n, number of participants with Parkinson's disease; N, total number of participants. All models are adjusted for age and sex.

## Discussion

In this population‐based cohort study we found that individuals with Parkinson's disease were more likely to have orthostatic hypotension. In contrast, the presence of orthostatic hypotension at baseline was not associated with increased risk of PD during long‐term follow‐up. Our findings suggest that orthostatic hypotension in the general population is not a prodromal marker of PD.

We hypothesized that the risk of PD would be higher in participants with orthostatic hypotension compared with those without orthostatic hypotension, especially in participants with neurogenic orthostatic hypotension. Our hypothesis was based on the observation that the prevalence of orthostatic hypotension in patients with PD is a relatively high 30% (range, 10%–65%)^6^ and increases with disease progression.^5^ However, our results did not support this hypothesis. Limiting the follow‐up duration to 5 and 10 years after the blood pressure measurements also did not show an increased risk of PD in participants with orthostatic hypotension or neurogenic orthostatic hypotension. The point estimates for neurogenic and early orthostatic hypotension even unexpectedly pointed in a contrary direction. However, the estimates of these subanalyses must be interpreted with caution because of the small number of participants with neurogenic and early orthostatic hypotension developing PD, which created large confidence intervals.

No other prospective population‐based study with continuous follow‐up has previously assessed the relation between orthostatic hypotension and Parkinson's disease. Yet, in contrast with our findings, some other studies did find an increased risk of PD in individuals with orthostatic hypotension.^9^, ^10^, ^11^, ^12^ One previous study applied the MDS criteria for prodromal PD to a population‐based setting in which the presence of PD was assessed at 3, 5, and 10 years.^11^ This study found that symptomatic orthostatic hypotension at baseline was associated with an increased risk of PD, although the number of patients with PD was small (n = 20).^11^ Furthermore, 1 large case–control study including 8166 patients with PD found a high increased risk of PD in hypotensive patients (RR, 3.23; 95% CI, 1.85–5.52).^12^ However, this study used a broad definition of hypotension including idiopathic hypotension and dizziness after standing up. Finally, 2 clinic‐based studies found that a substantial group of delayed or neurogenic orthostatic hypotension patients developed α‐synucleinopathies, including PD.^9^, ^10^ It should be noted, however, that these studies only included persons with orthostatic hypotension who were referred to a specialized center, thus probably representing individuals with more severe orthostatic hypotension than in the general population.

The pathophysiology of orthostatic hypotension in PD is incompletely understood.^19^ Several studies indicated that postganglionic sympathetic denervation in‐ and outside the heart with a loss of norepinephrine plays a role in the development of orthostatic hypotension in PD.^19^, ^20^, ^21^, ^22^ In addition, baroreflex failure and decreased renal sympathetic innervation have been described as causes of orthostatic hypotension in PD.^19^, ^20^, ^21^, ^22^ Although PD treatment can potentially further exacerbate orthostatic hypotension, this does not seem to be the primary cause of orthostatic hypotension, because the prevalence of orthostatic hypotension is also high in drug‐naive patients.^19^, ^20^ Deficiencies of the autonomic system have been described years before the onset of parkinsonism.^23^, ^24^ However, these deficiencies are not always accompanied directly by orthostatic hypotension or associated symptoms.^25^ It is thus plausible that despite the lack of an association between orthostatic hypotension and the risk of developing PD, the underlying pathophysiology causing orthostatic hypotension is already occurring years before the PD diagnosis.

Strengths of our study include our case‐finding approach with continuous and long‐term follow‐up for PD. In addition, we used a nonselected population of 55 years and older in which orthostatic hypotension was measured using a standardized protocol. Patients with PD in our study thus represent patients in the general population instead of patients presenting at specialized clinics. Finally, our study showed reliable estimates for the prevalence of orthostatic hypotension (19.8%), as our estimates were comparable to other population‐based studies using a similar definition of orthostatic hypotension.^7^ Our study was limited by the relatively low prevalence and incidence of Parkinson's disease, which could have influenced why we, contrary to expectations, did not find a significant association between prevalent PD and neurogenic orthostatic hypotension when using the ΔHR/ΔSBP ratio to determine neurogenic orthostatic hypotension. Also, the small number of participants with PD prohibited us from performing additional analyses into heart rate response or from stratifying for factors such as blood pressure medication or other vascular risk factors. Furthermore, the mean age of the study participants without PD at baseline was relatively high (68.9 years). As a result, we cannot rule out that an association between orthostatic hypotension and the risk of PD does exist in younger individuals. In addition, we measured orthostatic hypotension only once in this study. Thus, participants could have developed orthostatic hypotension after this measurement. An important final note is that we did not perform the Valsalva maneuver for our definition of neurogenic orthostatic hypotension. However, we used the ΔHR/ΔSBP ratio to separate neurogenic from nonneurogenic orthostatic hypotension, which was validated in a study of 402 participants with orthostatic hypotension and showed both high sensitivity (91.3%) and specificity (88.4%).^15^

## Conclusion

Our study suggests that orthostatic hypotension is common in patients with Parkinson's disease, but that orthostatic hypotension in the general elderly population is not associated with an increased risk of Parkinson's disease. These findings do not support the notion that orthostatic hypotension or neurogenic orthostatic hypotension is a prodromal marker of Parkinson's disease, contrary to what the current MDS criteria suggest. Future studies are warranted to replicate our findings, especially regarding the relation between neurogenic orthostatic hypotension and Parkinson's disease in a younger cohort.

## Author Roles

(1) Research project: A. Design and conceptualization, B. Data acquisition, C. Data analysis, D. Interpretation findings. (2) Manuscript: A. Writing first draft, B. Review and Critique.

L.J. Dommershuijsen: 1A, 1B, 1C, 1D, 2A.

A. Heshmatollah: 1A, 1B, 1C, 1D, 2A.

F.U.S. Mattace Raso: 1B, 2B.

P.J. Koudstaal: 1B, 2B.

M.A. Ikram: 1A, 1D, 2B.

M.K. Ikram: 1A, 1D, 2B.

## Financial Disclosures

L.J. Dommershuijsen: none.

A. Heshmatollah: none.

F.U.S. Mattace Raso: none.

P.J. Koudstaal: Royalties for *Textbook of Neurology*; Ed. Bohn Stafleu, Netherlands.

M.A. Ikram: Editorial Board of *Neuroepidemiology; Journal of Alzheimer Disease; European Journal of Clinical Investigation*; consultancy fpr Biogen Inc; Ministry of Public Health, Welfare, Sports Netherlands; and personal funding from EU ERC Starting Grant.

M.K. Ikram: funding from Stichting ParkinsonFonds.

## Supporting information


**Appendix S1**. Supporting Information

## Data Availability

The corresponding author has full access to all the data in the study and has final responsibility for the decision to submit for publication. Rotterdam Study data can be made available to interested researchers on request. Requests can be directed to data manager Frank J.A. van Rooij (f.vanrooij@erasmusmc.nl). Because of restrictions based on privacy regulations and informed consent of the participants, data cannot be made freely available in a public repository.

## References

[1] Heinzel S , Berg D , Gasser T , et al. Update of the MDS research criteria for prodromal Parkinson's disease. Mov Disord 2019;34(10):1464–1470. 31412427 10.1002/mds.27802

[2] Kalia LV , Lang AE . Parkinson's disease. Lancet 2015;386(9996):896–912. 25904081 10.1016/S0140-6736(14)61393-3

[3] Mahlknecht P , Seppi K , Poewe W . The concept of prodromal Parkinson's disease. J Parkinsons Dis 2015;5(4):681–697. 26485429 10.3233/JPD-150685 PMC4927924

[4] Postuma RB , Berg D . Advances in markers of prodromal Parkinson disease. Nat Rev Neurol 2016;12:622. 27786242 10.1038/nrneurol.2016.152

[5] Hiorth YH , Pedersen KF , Dalen I , Tysnes OB , Alves G . Orthostatic hypotension in Parkinson disease: a 7‐year prospective population‐based study. Neurology 2019;93(16):e1526–e1534. 31527282 10.1212/WNL.0000000000008314

[6] Velseboer DC , de Haan RJ , Wieling W , Goldstein DS , de Bie RMA . Prevalence of orthostatic hypotension in Parkinson's disease: a systematic review and meta‐analysis. Parkinsonism Relat Disord 2011;17(10):724–729. 21571570 10.1016/j.parkreldis.2011.04.016 PMC5199613

[7] Saedon NI , Pin Tan M , Frith J . The prevalence of orthostatic hypotension: a systematic review and meta‐analysis. J Gerontol A Biol Sci Med Sci 2018;75(1):117–122. 10.1093/gerona/gly188 PMC6909901 30169579

[8] Visser M , van Rooden SM , Verbaan D , Marinus J , Stiggelbout AM , van Hilten JJ . A comprehensive model of health‐related quality of life in Parkinson's disease. J Neurol 2008;255(10):1580–1587. 18821041 10.1007/s00415-008-0994-4

[9] Gibbons CH , Freeman R . Clinical implications of delayed orthostatic hypotension: a 10‐year follow‐up study. Neurology 2015;85(16):1362–1367. 26400576 10.1212/WNL.0000000000002030 PMC4626242

[10] Kaufmann H , Norcliffe‐Kaufmann L , Palma JA , et al. Natural history of pure autonomic failure: a United States prospective cohort. Ann Neurol 2017;81(2):287–297. 28093795 10.1002/ana.24877 PMC5323269

[11] Mahlknecht P , Gasperi A , Djamshidian A , et al. Performance of the movement disorders society criteria for prodromal Parkinson's disease: a population‐based 10‐year study. Mov Disord 2018;33(3):405–413. 29436728 10.1002/mds.27281

[12] Schrag A , Horsfall L , Walters K , Noyce A , Petersen I . Prediagnostic presentations of Parkinson's disease in primary care: a case‐control study. Lancet Neurol 2015;14(1):57–64. 25435387 10.1016/S1474-4422(14)70287-X

[13] Ikram MA , Brusselle G , Ghanbari M , et al. Objectives, design and main findings until 2020 from the Rotterdam Study. Eur J Epidemiol 2020;35(5):483–517. 32367290 10.1007/s10654-020-00640-5 PMC7250962

[14] EJo N . Consensus statement on the definition of orthostatic hypotension, pure autonomic failure, and multiple system atrophy. The Consensus Committee of the American Autonomic Society and the American Academy of Neurology. Neurology 1996;46(5):1470. 8628505 10.1212/wnl.46.5.1470

[15] Norcliffe‐Kaufmann L , Kaufmann H , Palma J‐A , et al. Orthostatic heart rate changes in patients with autonomic failure caused by neurodegenerative synucleinopathies. Annals of neurology 2018;83(3):522–531. 29405350 10.1002/ana.25170 PMC5867255

[16] Darweesh SK , Koudstaal PJ , Stricker BH , Hofman A , Ikram MA . Trends in the incidence of Parkinson disease in the general population: the Rotterdam Study. Am J Epidemiol 2016;183(11):1018–1026. 27188952 10.1093/aje/kwv271

[17] Clark TG , Altman DG , Stavola BLD . Quantification of the completeness of follow‐up. Lancet 2002;359(9314):1309–1310. 11965278 10.1016/s0140-6736(02)08272-7

[18] Gibbons CH , Schmidt P , Biaggioni I , et al. The recommendations of a consensus panel for the screening, diagnosis, and treatment of neurogenic orthostatic hypotension and associated supine hypertension. J Neurol 2017;264(8):1567–1582. 28050656 10.1007/s00415-016-8375-x PMC5533816

[19] Goldstein DS . Dysautonomia in Parkinson disease. Compr Physiol 2014;4(2):805–826. 24715569 10.1002/cphy.c130026 PMC4222515

[20] Fereshtehnejad S‐M , Lökk J . Orthostatic hypotension in patients with Parkinson's disease and atypical parkinsonism. Parkinsons Dis 2014;2014:475854. 24634790 10.1155/2014/475854 PMC3929346

[21] Sharabi Y , Goldstein DS . Mechanisms of orthostatic hypotension and supine hypertension in Parkinson disease. J Neurol Sci 2011;310(1–2):123–128. 21762927 10.1016/j.jns.2011.06.047 PMC4912223

[22] Coon EA , Cutsforth‐Gregory JK , Benarroch EE . Neuropathology of autonomic dysfunction in synucleinopathies. Mov Disord 2018;33(3):349–358. 29297596 10.1002/mds.27186

[23] Goldstein DS , Holmes C , Lopez GJ , Wu T , Sharabi Y . Cardiac sympathetic denervation predicts PD in at‐risk individuals. Parkinsonism Relat Disord 2018;52:90–93. 29032895 10.1016/j.parkreldis.2017.10.003 PMC6319357

[24] Goldstein DS , Sharabi Y , Karp BI , et al. Cardiac sympathetic denervation preceding motor signs in Parkinson disease. Clin Auton Res 2007;17(2):118–121. 17334896 10.1007/s10286-007-0396-1 PMC4615690

[25] Oka H , Toyoda C , Yogo M , Mochio S . Cardiovascular dysautonomia in de novo Parkinson's disease without orthostatic hypotension. Eur J Neurol 2011;18(2):286–292. 20602633 10.1111/j.1468-1331.2010.03135.x

