# Supplementary material for: Orthostatic Hypotension: A Prodromal Marker of Parkinson's Disease?
Source: Mov Disord. 2020 Sep 23;36(1):164–70. doi: 10.1002/mds.28303 (PMC7891584; doi:10.1002/mds.28303)

**Supplement**

**Supplemental table 1.** Proportion missing data imputed variables

| **Variable** | **Percentage missing** |
| --- | --- |
| Second systolic blood pressure measurement supine position | 0.02% |
| Second diastolic blood pressure measurement supine position | 0.02% |
| Heart rate measurement supine position | 0.24% |
| Heart rate measurement 1 minute | 0.75% |
| Heart rate measurement 2 minutes | 1.30% |
| Heart rate measurement 3 minutes | 2.21% |
| Heart rate measurement 4 minutes | 4.37% |
| Heart rate measurement 5 minutes | 5.11% |
| Systolic blood pressure measurement 1 minute | 2.37% |
| Systolic blood pressure measurement 2 minutes | 2.62% |
| Systolic blood pressure measurement 3 minutes | 3.41% |
| Systolic blood pressure measurement 4 minutes | 5.89% |
| Systolic blood pressure measurement 5 minutes | 6.37% |
| Diastolic blood pressure measurement 1 minute | 2.38% |
| Diastolic blood pressure measurement 2 minutes | 2.65% |
| Diastolic blood pressure measurement 3 minutes | 3.38% |
| Diastolic blood pressure measurement 4 minutes | 5.89% |
| Diastolic blood pressure measurement 5 minutes | 6.38% |

**Supplemental table 2.** Association of presence of Parkinson’s disease with orthostatic hypotension including additional criteria for participants with hypertension.

|  |  | Orthostatic hypotension | Neurogenic OH | Symptomatic OH | Early OH |
| --- | --- | --- | --- | --- | --- |
| **No prevalent Parkinson’s disease** |  |  |  |  |  |
| Number of OH participants / all participants |  | 862/6,237 | 435/6,237 | 108/6,237 | 580/6,237 |
| **Prevalent Parkinson’s disease** |  |  |  |  |  |
| Number of OH participants / all participants |  | 17/62 | 8/62 | 6/62 | 14/62 |
| Odds ratio (95% CI) |  | 1.65 (0.91 – 2.98) | 1.26 (0.59 – 2.72) | **3**.83 **(1**.57 **– 9.34)** | 1.71 (0.88 – 3.32) |

All models are adjusted for age and sex.

Abbreviations: CI; confidence interval, No; Number, OH; orthostatic hypotension.

**Supplemental table 3.** Orthostatic hypotension and risk of Parkinson’s disease including additional criteria for participants with hypertension.

|  |  | Parkinson’s disease | |
| --- | --- | --- | --- |
|  |  | n/N | Hazard ratio (95% CI) |
| No orthostatic hypotension |  | 106/5,375 | 1.0 [reference] |
| Orthostatic hypotension |  | 16/862 | 1.00 (0.58– 1.73) |
|  |  |  |  |
| No neurogenic orthostatic hypotension |  | 116/5,802 | 1.0 [reference] |
| Neurogenic orthostatic hypotension |  | 6/435 | 0.81 (0.32 – 2.04) |
|  |  |  |  |
| No early orthostatic hypotension |  | 113/5,657 | 1.0 [reference] |
| Early orthostatic hypotension (within 1 minute) |  | 9/580 | 0.87 (0.44 – 1.74) |
|  |  |  |  |
| No delayed orthostatic hypotension |  | 112/5,813 | 1.0 [reference] |
| Delayed orthostatic hypotension (after 3 minutes) |  | 10/424 | 1.23 (0.62 – 2.40) |

All models are adjusted for age and sex. n = number of PD, N = total number.

Abbreviations: CI; confidence interval.

**Supplemental Figure 1.** Kaplan-Meier curve with outcome death, stratified by orthostatic hypotension and neurogenic orthostatic hypotension status.


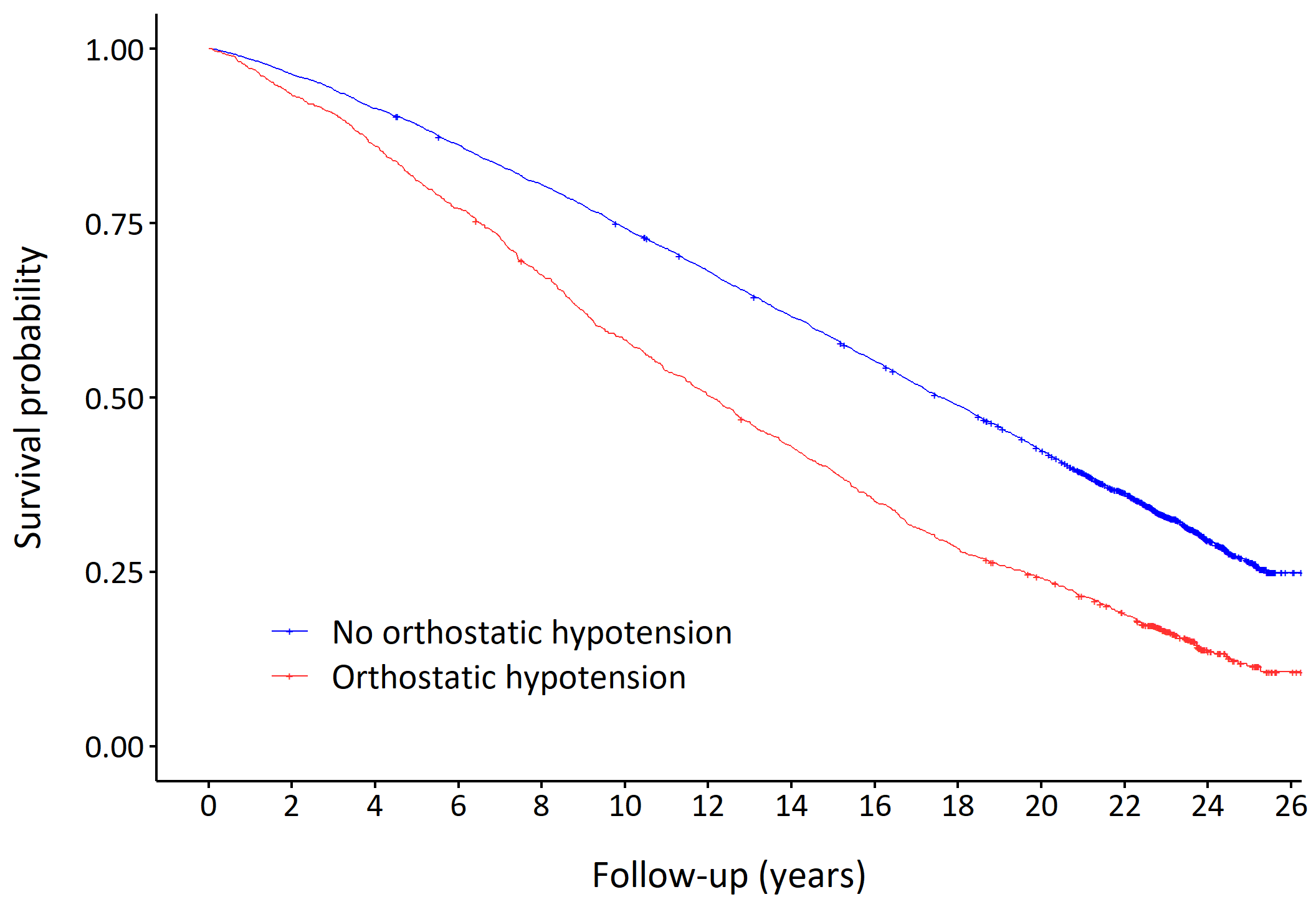


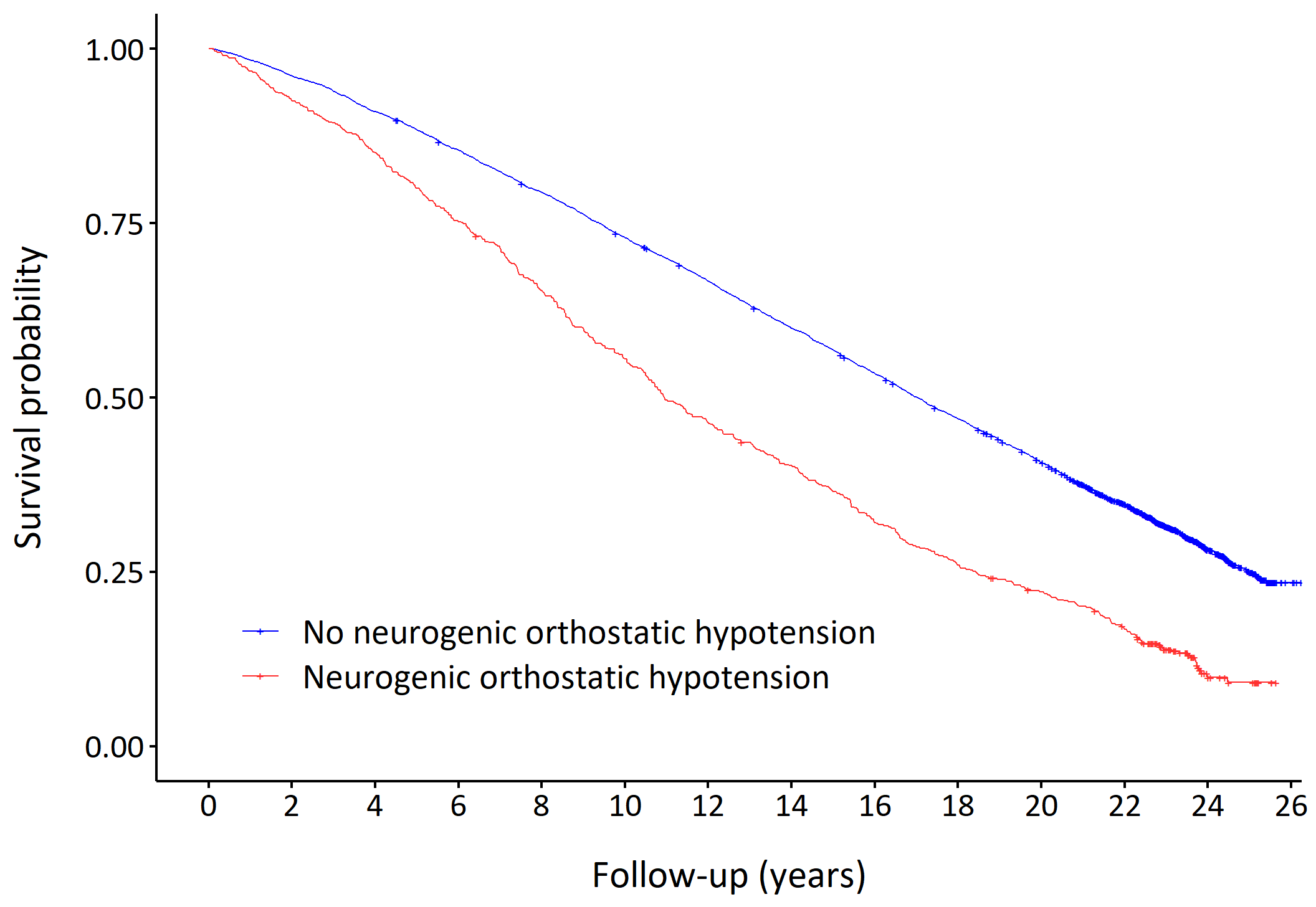

Supplement: Supplementary file 1 — Appendix S1. Supporting Information [file MDS-36-164-s001.docx]
